# P05.15. Online indexing service for research articles published on Ayurveda

**DOI:** 10.1186/1472-6882-12-S1-P375

**Published:** 2012-06-12

**Authors:** R Manohar, S Eranezhath, A Mahapatra, S Manohar R

**Affiliations:** 1 The Ayurvedic Trust, Coimbatore, India

## Purpose

Literature searching for prior research work on Ayurveda can turn out to be a frustrating exercise because the majority of the journals publishing papers on Ayurveda are not indexed in international databases such as Pubmed. Absence of controlled vocabulary that can facilitate literature search in the areas of Ayurvedic research makes it even more difficult to access available data effectively. An online indexing service exclusively for research articles published on Ayurveda has been set up to fill this gap.

## Methods

An online database called DHARA - Digital Helpline for Ayurveda Research Articles was launched to index articles published on Ayurveda in research journals worldwide (http://www.dharaonline.org). Research journals of Ayurveda, CAM, biomedicine as well as related fields like medical anthropology and medical sociology were screened to index research papers dealing with Ayurveda. Depending on each journal’s policy for public access, titles, abstracts and links to full text have been provided. The database is continuously being updated.

## Results

Over 6000 papers covering more than 700 research journals worldwide have been indexed in the DHARA database. Keyword searches, advanced searches with boolean operators and limiting searches with controlled vocabulary are the key features of the database. Option to search with field tags has also been provided. Author and journal indices provide additional information about authors and journals, as well as serve to assist in to retrieval of research papers related to specific authors or journals.

## Conclusion

The DHARA database makes it possible for the first time to obtain a high altitude view about research in the field of Ayurveda. It also makes it possible to review prior research work, identify gaps and prioritize future research effectively. Wider publicity for DHARA database, which is a free service, will facilitate worldwide access to Ayurvedic research.

